# National and State Boards of Health

**Published:** 1879-09

**Authors:** 


### NATIONAL AND STATE BOARDS OF HEALTH.

It is pretty generally known that the last congress not only appointed a "National Board of Health," of which Prof H. A. Johnson, of this city, is a member, but half a million of dollars was appropriated and placed at the disposal of said board.

The law creating the national board contemplates its direct co-operation with state boards of health, wherever such exist, and with such other state quarantine and sanitary authorities as may have been established. It is desirable that all our readers should be well informed in regard to the relations between the national and state boards, particularly in regard to the expenditure of money and the obtaining of supplies. Hence we copy the following in full, from the *National Board of Health Bulletin*, for August 16 :

### NATIONAL CO-OPERATION WITH STATE AND LOCAL BOARDS.

Although the propor method of securing the co-operation of the national board of health with state and local boards, in preventing the spread of yellow fever from one State into another has heretofore been published and widely circulated, it happens almost daily that applications are received at the office of the national board which require to be returned to the parties making them, by reason of their failure to comply with the conditions under which the board is authorized to grant the desired aid. It is accordingly found to be necessary again to call attention to certain points which should be observed by state and municipal authorities in making application for aid and co-operation. To this end the following circular has been prepared and is now published.

### CIRCULAR NO. 7.

The following rules govern the action of the national board of health at present in aiding State and local boards to enforce regulations of such boards preventing the introduction of contagious and infectious diseases into the United States, or into any one State from another.

1. The regulations to be enforced are those of State and local boards, whether adopted at the recommendation of the national board or otherwise, and not those of the national board. The national board has recommended certain regulations for adoption by State and local boards. Up to the present time these recommendations have been adopted by the following boards, viz.:

The State boards of Arkansas, Illinois, Kentucky, Louisiana, Mississippi, New Jersey, North Carolina and Tennessee; the local boards of Brunswick, Ga., Brownsville, Tex., Bayou Sara, La., Cairo, Ill., Carlinsville, Ill., Decatur, Ala., Delhi, La., Fernandina, Fla., Huntingdon, Tenn., Jacksonville, Fla., Lauderdale County, Mississippi, Meridian, Miss., Mobile, Ala., Pensacola, Fla., Shelbyville, Tenn., Saint Louis, Mo., Tampa, Fla., Vicksburg, Miss.

The regulations of the above named boards are therefore to a certain extent uniform, and approved by the national board, and therefore are such as it will aid in enforcing, when necessary.

State and local boards which have not adopted the recommendations of the national board are requested to do so as soon as convenient, in order to secure uniformity of action.

It should be observed that these recommendations are for a minimum amount of precaution, and therefore that additional precautions may be taken by state or local boards if deemed necessary, it being borne in mind all the while that the end in view is to secure or restore the public health by measures which interfere with travel or traffic as little as possible; in other words to render commerce *secure;* and (with rare exceptions) *not to put an end to, or even suspend it.* In this connection it is proper to add that non-intercourse quarantines, especially by *local* authorities, are not approved by this board.

2. Applications to the national board of health for aid should be made by or through the state board; or in case there is no State board, then by or through the governor of the State.

3. Application for aid should give *details* of what is required, and the estimated cost for each item. Amongst other things should be specified the duties and powers of the officers whose appointment or payment is requested.

4. The application should be accompanied by an official certificate from the governor of the State or the mayor or other chief officer of the municipality, respectively, to the effect that there are no state or municipal funds available to carry out the particular sanitary measures because of which the application is made. Official information should be given therein of the adoption by such state or local board of any rules and regulations that have been recommended in such case by this board, and of any other state or local rules and regulations that appear to be necessary for the purpose in question.

5. Of the supplies required for the sick those furnished by this board to local authorities shall, as a general rule, be applied to other objects than those of shelter and furniture, which should be furnished by such authorities. Where however it shall be otherwise ordered the local authorities will be expected to account to this board from time to time for the safe-keeping and proper use of the furniture, provisions, medicines, etc., so furnished.

6. Whenever this board shall order the erection of temporary buildings, or provide any buildings for the purpose of quarantine the necessary contracts therefor shall be made by one of its own officers or agents, subject to the approval of the board or of its executive committee.

7. Care should be taken that the officers to be paid from funds furnished by the national board are employed only in such number and for such time as there is actual need of their services. The national board of health reserves the right of judging from time to time, by means of reports received from its own agents, whether such need exists.

8. Funds are not furnished by the treasury to state or local boards. They are placed in the hands of the disbursing clerk of the national board of health, by whom bills, properly certified and approved, will be paid by check on Washington or New York. All bills must be in accordance with the estimates as approved by the secretary of the treasury, must be made out in duplicate on forms furnished by the national board, and be *certified*, as to their correctness, by some authorized officer of the state board or by the governor of the state and must be approved by some member or special inspector of the national board duly authorized.

20

The names of all persons whose services as inspectors, etc., are to be paid for out of its funds must previously to their appointment be submitted to and approved by the national board.

It is expected that at the close of the season a full report will be made by state boards of health to the national board as to their operations in carrying out those rules and regulations for the prevention of the spread of yellow fever, in which the national board has rendered aid and co-operation, and it is desired that copies of all orders issued from time to time to inspectors shall be promptly furnished to this board.

It is to be remembered that a full account of its expenditures must be made by the national board of health to congress, and that such account ought to set forth these expenditures in detail, and exhibit their propriety and necessity.

It is therefore essential that state and municipal boards shall co-operate with the national board in supplying material for such an account, and it is earnestly desired that they will preserve and furnish due evidence of the propriety of each item of their expenditure for both persons employed and articles purchased with the funds in question; particularly as the future aid of both state and national boards must depend largely upon the record for efficiency and economy made during the year now current.

